# Understanding the impact of m*-*learning platform LEAP on learning outcomes and health care referral behavior of community health volunteers in Kenya

**DOI:** 10.1093/oodh/oqae036

**Published:** 2024-12-02

**Authors:** John Harnisher, Anzhelika Lyubenko, Peter Kisare Otieno

**Affiliations:** Research, DataKind, New York City, NY, USA; Data Science, DataKind, Des Moines, IA, USA; Health Innovations, Amref, Nairobi, Kenya

**Keywords:** community health volunteers, m-learning, LEAP, mobile health, Kenya, data science

## Abstract

Mobile learning (m-learning) platforms are increasingly used to train healthcare workers as a strategy to address the global healthcare worker shortage. These platforms are also attractive because they are low-cost and accessible to anyone with a phone, providing the potential to foster equitable health information in the world’s most remote and under-resourced areas. Because of this opportunity, many health initiatives have deployed m-learning approaches to meet their humanitarian goals, yet studies of their implementation are scattered. We provide a case study example of how a non-profit partnership between Amref and DataKind was leveraged to more robustly assess the data of the m-learning platform LEAP in Kenya, leading to a more in-depth understanding of its functionality and impact. These types of assessments are crucial to building data-informed decision making that can effectively advance the use of digital technologies for healthcare. The main findings from this work are as follows: (i) investment in analytics infrastructure is critical, (ii) structured m-learning programs have better outcomes, (iii) practicals are the most common activity, (iv) scores and completion rates are higher for learners that use the program in English and (v) referrals to health care facilities increased after formal LEAP programs.

**RESUMEN:**

Las plataformas de aprendizaje electrónico móvil (m-learning) están siendo usadas con cada vez más frecuencia en la capacitación de prestadores de salud, como una estrategia para enfrentar la escasez mundial de prestadores de salud. Estas plataformas son además atractivas por ser de bajo costo y fácil acceso para cualquier persona con un teléfono celular, haciendo posible una más equitativa difusión de la información médica, incluso en las zonas más remotas y con menos recursos del mundo. Dadas estas oportunidades, muchas iniciativas sanitarias han desplegado planteamientos de aprendizaje móvil para alcanzar sus metas humanitarias, pero los estudios acerca de su implementación son algo dispersos. Presentamos aquí un ejemplo de estudio de caso de cómo una colaboración sin fines de lucro entre Amref y DataKind fue apalancada financieramente para poder evaluar de manera más robusta los datos de la plataforma de m-learning LEAP en Kenia, llevando a una comprensión más profunda de su funcionalidad e impacto. Este tipo de evaluaciones son cruciales para construir sistemas de toma de decisiones basadas en datos, que puedan avanzar de manera efectiva el uso de tecnologías digitales en el cuidado de la salud. Los hallazgos principales de este trabajo son: 1) la inversión en infraestructura analítica es crítica, 2) los programas de m-learning estructurados tienen mejores resultados, 3) las prácticas son la actividad más común, 4) las calificaciones y tasas de finalización son mayores para los aprendices que usan el programa en inglés, y 5) las referencias de pacientes a instalaciones de salud incrementaron tras el uso formal de los programas LEAP.

**RESUMO:**

As plataformas de aprendizagem móvel (m-learning) são cada vez mais utilizadas para formar profissionais de saúde como estratégia para fazer face à escassez global de profissionais de saúde. Estas plataformas são também atrativas por serem de baixo custo e acessíveis a qualquer pessoa com um telemóvel, proporcionando o potencial para promover informação de saúde equitativa nas áreas mais remotas e com menos recursos do mundo. Devido a esta oportunidade, muitas iniciativas no domínio da saúde têm utilizado abordagens de aprendizagem móvel para atingir os seus objetivos humanitários, mas os estudos sobre a sua implementação são dispersos. Apresentamos um exemplo de estudo de caso de como uma parceria sem fins lucrativos entre a Amref e a DataKind foi aproveitada para avaliar de forma mais sólida os dados da plataforma de aprendizagem móvel LEAP no Quénia, levando a uma compreensão mais aprofundada da sua funcionalidade e impacto. Estes tipos de avaliações são cruciais para a tomada de decisões informadas por dados que possam efetivamente fazer avançar a utilização das tecnologias digitais nos cuidados de saúde. As principais conclusões deste trabalho são as seguintes: 1) o investimento em infraestruturas analíticas é fundamental; 2) os programas estruturados de aprendizagem móvel têm melhores resultados; 3) as atividades práticas são as mais comuns; 4) as pontuações e as taxas de conclusão são mais elevadas para os alunos que utilizam o programa em inglês; e 5) as referências a instalações de cuidados de saúde aumentaram após os programas formais LEAP.

**RÉSUMÉ:**

Les plateformes d’apprentissage mobile (m-learning) sont de plus en plus utilisées pour former les professionnels de la santé comme stratégie pour faire face à la pénurie mondiale de personnels de santé. Ces plateformes sont également attrayantes car elles sont peu coûteuses et accessibles à toute personne disposant d’un téléphone, offrant ainsi la possibilité de favoriser une information sanitaire équitable dans les zones les plus reculées et les moins dotées en ressources du monde. En raison de cette opportunité, de nombreuses initiatives en matière de santé ont déployé des approches d’apprentissage mobile pour atteindre leurs objectifs humanitaires, mais les études sur leur mise en œuvre sont dispersées. Nous fournissons un exemple d’étude de cas sur la manière dont un partenariat à but non lucratif entre Amref et DataKind a été exploité pour évaluer de manière plus fiable les données de la plateforme d’apprentissage mobile LEAP au Kenya, conduisant à une compréhension plus approfondie de sa fonctionnalité et de son impact. Ces types d’évaluations sont essentiels pour établir une prise de décision fondée sur des données qui peut faire progresser efficacement l’utilisation des technologies numériques dans les soins de santé. Les principales conclusions de ce travail sont les suivantes: 1) l’investissement dans les infrastructures d’analyse est essentiel, 2) les programmes d’apprentissage mobile structurés ont de meilleurs résultats, 3) les travaux pratiques sont l’activité la plus courante, 4) les scores et les taux d’achèvement sont plus élevés pour les apprenants qui utilisent le programme en anglais, et 5) les références aux établissements de soins de santé ont augmenté après les programmes LEAP formels.

## INTRODUCTION

The last decade has seen ever growing interest in using mobile technologies to aid health initiatives across the world, referred to collectively as mobile health or m-health [1–3]. While m-health initiatives span from telemedicine, to mobile phone push notifications of public health messages, to patient monitoring devices [3], mobile learning (m-learning) platforms to aid in community health training are of particular recent interest [4].

Utilizing m-learning approaches offers the opportunity to address two key issues within healthcare—(i) a global shortage of health workers that is projected to be most acute across Africa and Eastern Mediterranean regions [5], and (ii) inequitable access to training in low- and middle-income countries [4]. Training community health workers (CHWs), who are vital in promoting health initiatives by providing basic health assessment and treatment, and connecting communities to formal services, is one way to shore up the health worker gap (Braun et al., 2013); [6]). However, it is crucial that CHWs receive proper training, and since they work as lay volunteers, training modalities must fit their lives and the needs of the often remote communities they work with, including cost and accessibility considerations (Braun et al., 2013); [7].

Mobile learning has been championed as a way to meet these needs, increasingly used to train CHWs to address the health worker gap and to reflect their unique role and needs [4, 6]. The main argument for the benefit of m-learning is that it is learner centric and seen as a vehicle for accessible and adaptable life-long learning [8]. Since m-learning is an emerging space, it generally has a lack of underlying pedagogical theory, but some have tied it to the various learning theories of behaviorism, constructivism, and situated learning, all of which emphasize the importance of learner and context specific modes of knowledge construction [8]. For a full historical review of the development of m-learning, see Crompton [9].

Ultimately, many find that m-learning provides educational opportunities that are more flexible, self-directed, informal and guided by personalized needs and interests [10]. Further, m-learning is often more low-cost and accessible, available to anyone with a phone, allowing important health information to have a broader reach (e.g. [11]). There are nonetheless some disadvantages or risks to m-learning such as the potential to be distracting, inefficient, create uncertainty around credibility of information, and even create risks around using technology inappropriately around patients [12]. In addition there are technological limitations—the cost of mobile devices can be prohibitive, the small screens can be inaccessible for users, connection speed can hinder content delivery, and information can be overloading for some learners [13].

Despite drawbacks, the positive features of m-learning have made exploring its use in the health context a key priority for many health organizations (e.g. WHO, 2020). However, the evidence base for m-learning in these contexts, particularly for training community health workers, is not well developed [4]. The following case-study aims to contribute to this gap. It provides an example of how a partnership between a humanitarian organization and a data science organization was leveraged to more quantitatively analyze the process and outcomes of a heavily utilized m-learning platform, with lessons for application and development. While the exploratory findings from this analysis provide important insights, a critical outcome is the establishment of a pathway for future analysis, built on foundational work to merge disparate databases.

### Case study description—a partnership between Amref and DataKind

In 2022, Amref, a non-profit targeting improved and sustained health access in Africa, partnered with DataKind, a data science non-profit, to advance their understanding of their long-running m-learning platform called LEAP. Amref has been actively working to improve the health worker gap in Africa since 1974, reaching more than 13 million individuals, including CHWs, Community Health Volunteers (CHVs) and policy makers [14]. They deployed LEAP over a decade ago with the goal of increasing and improving health worker training, especially in more remote or hard to access areas. LEAP is a telephone application that uses Interactive Voice Recognition (IVR) and Short Message Service (SMS) to train community health volunteers on key health-related topics using Ministry of Health approved curricula. LEAP programming can be either structured (i.e. part of a comprehensive series of topics that lead to specific outcomes) or unstructured (i.e. ad hoc information). Engagement points within LEAP include exposure to the content, quizzes and practical activities (i.e. scenario based assessments).

LEAP has been widely used in Kenya, training more than 57 076 CHWs, who have then provided essential health education to individuals and households on topics including health education, first aid, nutrition and referral services [15]. Kenya decentralized its healthcare system in 2013, further devolving authority to the county level for administering all health care services. While there were benefits to this shift, it also came with challenges such as inadequate funding and staffing, which has resulted in worker strikes and inequitable distribution of healthcare workers [16]. Such conditions contribute to why training more CHW and CHVs is so critical to the work of Amref in Kenya.

While LEAP has been used for years with strong anecdotal evidence of success [17], the program is lacking more rigorous analysis of its processes and outcomes to inform its continued use. This is an issue many interventions within the m-health and m-learning space have experienced, leading to ambivalent empirical evidence and hindering scalability [2]. LEAP has also been donor funded, with no long term contracts to support its expansion or sustainability. Thus, to continue the work of LEAP, more analysis is necessary to support its next phase of development and understand its progress towards its aims. This analytical need is where DataKind entered the space, offering pro-bono data science expertise supported by grants to explore the implementation of LEAP by building vital data infrastructure and embarking on initial exploratory analysis.

## METHODS AND APPROACH

DataKind evaluated quantitative data on LEAP learner characteristics and platform engagement (see Appendix for variable list) to explore the overall effectiveness of LEAP training, focusing on descriptive statistical analysis (e.g. average completion rates by learner attributes, average topics completed, average time taken to complete topics, etc.) The scope of this collaboration and limits of the data did not allow for more robust analysis such as inferential statistics or testing for statistical significance, but rather was the beginning of a data analytics process to assist AMREF in setting up their data so that it is ready for more intensive analysis in the future. To be clear, this analysis used in this case study is not designed to allow causal interpretation of the results, only suggestions of trends and potential impacts. As such, the evidence of the initial assessment provided in the following section is exploratory in nature, providing suggestions of where to extend future work.

The analysis had two main components**—**an outcome assessment and an implementation assessment. The goal of the outcome assessment was to explore the potential connection between the learning in the LEAP system to external behaviors of community health volunteers, specifically in terms of referral behavior. The goal of the implementation assessment was to investigate how LEAP fits into the working ecosystem for health care workers and how it might be better optimized. Understanding Amref’s ultimate goal of improving community health outcomes was not within the scope of this analysis, largely due to data limitations. Table 1 summarizes the key analytical questions asked of the data for this project, oriented around implementation and outcomes.

**Table 1:** Assessment questions

Main question—Does LEAP usage lead to improved knowledge for community health volunteers (CHVs)?	
Assessment Area	Sub Questions
*Implementation*—understanding what is happening within the LEAP platform	Is CHV learning effective on the platform? Are there differences on topic performance? Are all the functionalities of LEAP used? Can we segment learners based on behavior? How much time does it take to progress through topics? Can we predict future performance based on key attributes?
*Outcome—*behavioral impact of learning in LEAP	Does CHV training within LEAP lead to increased health care referrals targeted to topics reviewed in LEAP?

The scope of this investigation was limited to 5 years of LEAP data for community health volunteers (2018–2022). This sample of LEAP data was used to analyze completion rates and average LEAP scores across all topics and programs during this time period. LEAP data are automatically collected from the platform itself via logs of user interactions. Then, to understand LEAP data differentiated by learner characteristics and its connection to referral behavior, it was merged with m-Jali database information, which covers a shorter time frame (2020–2022) and fewer CHVs. The m-Jali database is a community health engagement platform where CHWs and households report local level health data, including demographics and referrals, the primary proxy for the impact of LEAP for this analysis. As such, the m-Jali database data are user submitted.

There is not a one-to-one relationship between the LEAP data and the m-Jali data, and the LEAP platform design does not support generalized analysis across all initiatives because internal data are organized according to specific community health programs. Thus, the process to merge them, using a Python script, resulted in an additional subsample, and depending on the variable assessed (e.g. gender, language), there were additional gaps, resulting in different sample sizes for different findings. Ultimately, because the datasets were disparate, covering different timeframes and variables, merging LEAP and m-Jali data limited additional analysis (i.e. synthesizing program outcomes by language, gender, etc.) to just two structured LEAP programs that could be compared to overall unstructured programs. These were the only two programs that had complete, merged data between m-Jali and LEAP:

- Innovative Partnership for Universal Sustainable Healthcare (iPUSH)—provides training in areas such as maternal and child health, family planning, HIV/AIDS prevention and treatment and nutrition (specific topics within data are Danger Signs, Water Safety, Health Promotion, Maternal and Child Health and Universal Health Coverage)
- The Blueprint for Innovative Healthcare Access—a partnership model focused on delivery and implementation, and is applied in a targeted and measurable way to save and improve the lives of patients with cancer and other Non-Communicable Diseases (specific topics within data are Hypertension, Cancer and Diabetes)

### Findings

#### *Case study population*—*LEAP learners in Kenya*

The sample of LEAP data alone contained 485 116 learners. After merging m-Jali data with LEAP data, the data set narrowed to 78 872 learners. This subsample of LEAP community health volunteer learners was less male and less Swahili-speaking than the general population of Kenya (see Table 2). The role these differential population characteristics plays in learner outcomes was not assessed in this analysis because of its exploratory nature, but could prove to be an important element for future investigations.

**Table 2:** Population demographics: LEAP learners vs. Kenyan National Statistics

*Attribute*	*LEAP learners (n = 78 872)*	*Kenya National Statistics*
%Male	30%	49.7% [18]
%Swahili speaking	17%	~80% [19]

**Table 3:** Average LEAP scores for structured (iPUSH and Blueprint) vs. Other programs

*Program*	*Average score (out of 100)* *(n = 78 872)*
iPush	56
Blueprint	49
Other (non-structured)	20

#### Implementation

The implementation assessment revealed a number of useful insights. Across the initial LEAP data sample, the completion rate for learners was 38% (n = 485 116). Approximately, 8% of learners received scores for iPUSH programs, 7% for Blueprint programs and 85% for other programs (n = 220 643). For the subset of LEAP data connected to m-Jali demographics (n = 78 872), completion rates were similar between male (36%) and female (38%) learners, but they were higher for English learners (40%) versus Swahili learners (26%). Average LEAP scores were also higher for English learners (50) versus Swahili learners (42) (score out of 100, n = 78 872). These findings suggest a need to explore the role of language as a potential barrier to using LEAP.

**Figure 1:**
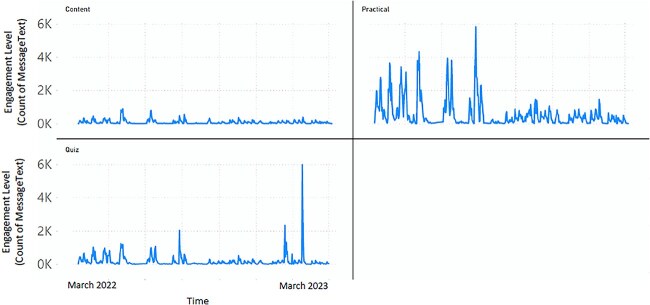
LEAP platform engagement by type (content, quiz, practical) and time

There were also important distinctions in how learners interacted with the content based on the type of program and its features, which can be used to experiment with optimization of the program to increase engagement. For instance, the more structured programs of iPUSH and Blueprint had higher average LEAP scores than non-structured programs (see Table 3) (iPUSH = 56, Blueprint = 49 vs. others = 20 [score out of 100, n = 78 872]). In addition, practical learning elements had higher levels of engagement (as measured by outgoing SMS messages) than content or quizzes (Fig. 1) combined, these data points suggest that emphasizing structured programs and providing them with multiple practical learning elements could foster higher engagement and completion.

**Table 4:** Average leap scores by topic within programs and by gender

*Program*	*Topic*	*Average score (out of 100)* *(n = 78 872)*	*Average score female (n = 23 994)*	*Average score male (n = 54 789)*	*Average score gender not identified (n = 89)*
Blueprint	Hypertension	55	57	51	67
Blueprint	Cancer	46	46	47	-
Blueprint	Diabetes	45	47	42	-
iPUSH	Danger signs	61	61	62	63
iPUSH	Water safety	61	61	62	67
iPUSH	Health Promotion	58	58	57	57
iPUSH	Maternal and child health	53	56	46	63
iPUSH	Universal health coverage	44	44	42	-
Other	-	20	19	19	50

**Figure 2:**
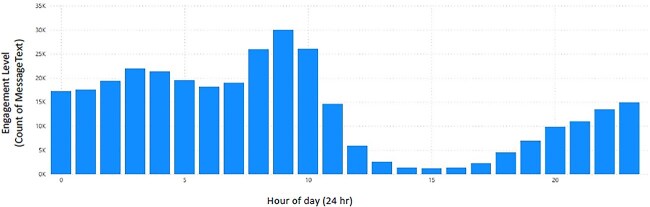
LEAP platform engagement by time of day

Assessment of learner activity trends found that engagement was highest on Tuesdays and Wednesdays, and there was activity throughout the day with lulls at meal times (see Fig. 2). In terms of platform type, use was largely even between IVR (48%) and SMS (52%) modalities across time for a sample of data (n = 3032) (For this analysis, data were only available for four quarters in 2022 and one quarter in 2023). This suggests that the method of m-learning may not be a large factor in engagement, but timing reminders or program promotions to certain times of the week could be helpful. Finally, learners completed their training within a week of engaging with a topic***—***the maximum average time spent for a topic was 103 h, with a total iPUSH average of 63 h and a total Blueprint average of 26 hrs. This indicates a week is sufficient time and can be used as a benchmark for planning future programs and managing reminders. While this section has provided an overview of key takeaways, Tables 2-5 provide additional specific outcome data details.

**Table 5:** Average LEAP scores per topic by language

*Program*	*Topic*	*English average score (out of 100)* *(n = 419 553)*	*Swahili average score (out of 100)* *(n = 65 532)*
Blueprint	Hypertension	56	50
Blueprint	Cancer	48	15
Blueprint	Diabetes	44	59
iPUSH	Danger signs	63	54
iPUSH	Water safety	62	53
iPUSH	Health promotion	59	51
iPUSH	Maternal and child health	53	54
iPUSH	Universal health coverage	45	36
Other	Other	22	10

#### Outcomes

**Figure 3:**
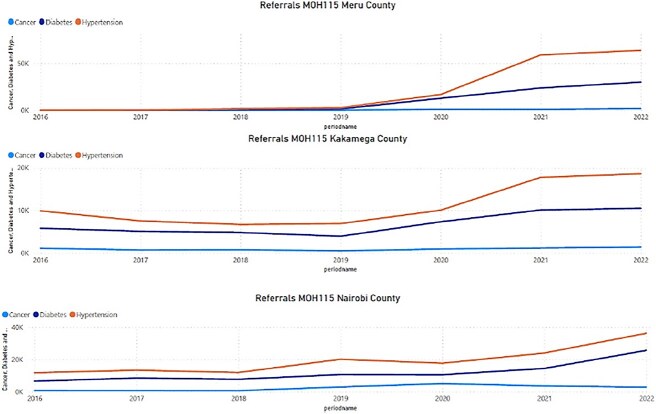
Total referrals across counties over time for blueprint program

The main way impact was operationalized for this exploratory assessment was via referral behavior. While causal connections and establishment of statistical significance are not possible, the assessment revealed a potential increase in referrals after the Blueprint program in particular (Note: data not available across time for i-PUSH to make an assessment of referrals before/after) (see Fig. 3, note that the Blueprint program for the topics addressed was initiated in 2019). Even though only small percentages of learners made referrals after these programs (9% for Blueprint and 1% for iPUSH), each learner made an average of 12 referrals. Referral behavior was steady across LEAP scores for Blueprint learners, but was inconsistent across LEAP scores for iPUSH learners, suggesting a potential differentiation in learner referral preference that is unclear from this level of analysis. Future research should investigate this potential further by exploring additional data sources that can foster more rigorous statistical analysis to better understand the links between LEAP and learner behavioral impacts.

## CONCLUSION

At the completion of the project, the analysis was able to provide valuable results regarding usage and overall learning within the platform, which can guide adjustments to the how LEAP is implemented. Results indicated that structured programs tended to yield higher overall learning scores and higher completion rates for learning topics than did unstructured learning. In addition, insights on how learning might be differentiated by gender and language can help further refine how LEAP can adjust in the future. There were also encouraging findings related to overall engagement and completion rates, which is especially valuable given the persistent difficulty of reaching community health volunteers through traditional learning settings.

As is common with learning platforms, connecting learning data to behavioral outcomes was not easily accomplished. An initial connection between the learning platform and the outcome data in terms of referrals indicated a potential association between learning and referrals. More investigation is necessary to understand this connection and if any true causal linkages exist. Experimentation in this area, such as comparing the LEAP learners to non-LEAP learners, could lead to a more direct link between learning activity and behavior change. While such conclusions are not possible within this case study, the establishment of a data infrastructure that connects learning data to referrals is an incredibly powerful step in the right direction for future work to connect LEAP outcomes and community health.

In addition to the findings of the analytical assessment, the process of this case study revealed numerous procedural insights oriented around data systems. For instance, the databases for m-Jali and LEAP are designed for end-user software and do not capture analytics. The data linkages between m-Jali and LEAP were also challenging when trying to conduct an assessment because those linkages were sporadic and dependent on specific programs versus continuous across all programs, as seen in the shifting subsample sizes throughout the data. Further, data quality issues like categorical variable categories changing over time made linkages even more difficult, and manual extractions of additional data sets were needed to provide a full analysis.

These kinds of data management insights are just as valuable as any insights about potential impact of LEAP on CHWs because more robust analysis cannot be conducted without a solid data systems foundation. This is a finding that can benefit any initiative, illustrating the importance of investing in data infrastructure that can better serve assessment goals. The LEAP learning platform was created 10 years ago and would benefit from further guidance regarding updating data collection and storage processes to align them with more current platform approaches. Such updates would allow future analysis to link m-learning more concretely to potential community health outcomes, allowing deeper understanding of the role this specific training modality has in this context and thus how it can be changed to increase its impact and accomplish the very crucial need of increasing the number of well-trained CHWs across the world. Ultimately, this case study highlights the importance of collaborations like the one between Amref and DataKind in setting up the foundations for robust assessments of popular m-learning tools within the health care worker context, a vital step in the direction to understanding their impact and how they can better serve their communities.

## Supplementary Material

Appendix_-_Publication_Version_oqae036

## Data Availability

Access to both LEAP and m-Jali data sources is managed by AMREF, and DataKind was given access to the data for the analysis of this case study with permission to publish in summary reporting format. We do not have permission to share the raw data underlying this analysis.
