# Supplementary material for: Understanding the impact of m-learning platform LEAP on learning outcomes and health care referral behavior of community health volunteers in Kenya
Source: Oxf Open Digit Health. 2024 Dec 2;2(Suppl 2):ii25–31. doi: 10.1093/oodh/oqae036 (PMC11936318; doi:10.1093/oodh/oqae036)
Supplement: Appendix_-_Publication_Version_oqae036 [file Appendix_-_Publication_Version_oqae036.docx]

***Supplementary Materials***

**Understanding the impact of m-learning platform LEAP on learning outcomes and health care referral behavior of Community Health Volunteers in Kenya**

**John Harnisher*^1^, Anzhelika Lyubenko,^2^ Peter Kisare Otieno^3^**

**1 Research, DataKind, New York City, New York, USA**

**2 Data Science, DataKind, Des Moines, Iowa, USA**

**3 Health Innovations, Amref, Nairobi, Kenya**

**Correspondence:**

***John Harnisher**

[**john.harnisher@datakind.org**](mailto:john.harnisher@datakind.org)

**DataKind, Inc.**

**271 Cadman Plaza E. Unit 24554.**

**Brooklyn, NY 11202-8332**

**Appendix**

**Variable list**

- LEAP data in Kenya:
  - Learner details: name, address (village), age, gender, nationality, Community Unit (CU), Link Facility, County, Sub county (Kenyan Context); When was your last training session, Number of Years as a CHW, Number of Households Manned, Learner Level of Education; type of phone used (basic or smart);
  - All learning activities by all learners (topics accessed; SMS/IVR received and sent; group chat; job aid consulted; Decision Trees, Pre-Tests and Post Tests,
  - Learning performance per learner per topic, completion performance per topic
  - End-of-topic survey answers raw data (linked to learner)
  - Group chat raw data (linked to learner)
  - Open-ended questions raw data (linked to learner)
  - Supervisor (CHEW) data: name, address, age, gender, nationality, learners (CHW) supervised, Community Units Supervised, TOT Model
  - Supervisor (CHEW) activity data (SMSs sent to learners)
- m-Jali variables merged with LEAP data:
  - New and old referrals
